# Compliance with different physical activity recommendations and its association with socio-demographic characteristics using an objective measure

**DOI:** 10.1186/1471-2458-13-136

**Published:** 2013-02-14

**Authors:** Tineke Scheers, Renaat Philippaerts, Johan Lefevre

**Affiliations:** 1 Department of Kinesiology, KU Leuven, Tervuursevest 101, Leuven, 3001, Belgium; 2 Research Foundation - Flanders, Brussels, Belgium; 3 Department of Movement and Sport Sciences, Ghent University, Watersportlaan 2, Gent, 9000, Belgium

**Keywords:** Prevalence of physical activity, SenseWear Armband, Objective monitoring, Public health guidelines, Epidemiology

## Abstract

**Background:**

In the past decades, several public health guidelines concerning physical activity have been published. This study evaluated compliance with various physical activity guidelines and examined the associations between meeting the guidelines and socio-demographic characteristics.

**Methods:**

Data were obtained from 357 Flemish men and women (41.9 ± 9.6 years). Physical activity was assessed for seven consecutive days using the SenseWear Armband. The prevalence of sufficient physical activity was calculated according to various public health guidelines. Logistic regressions examined the associations between socio-demographic characteristics and the odds of meeting the different guidelines.

**Results:**

87.2% of men and 68.1% of women achieved ≥150 min/week of moderate-to-vigorous physical activity (MVPA), but only 57.6% and 37.3% accumulated this amount as ≥30 min/day on ≥5 days/week. With regard to vigorous physical activity, 27.9% of men and 15.7% of women achieved ≥75 min/week and 12.8% and 7.0% achieved ≥20 min/day on ≥3 days/week. In addition, 34.9% of men and 21.6% of women attained an average physical activity level (PAL) of 1.75 MET and thus met the criteria for weight maintenance. Only 16.3% of men and 14.1% of women took 10000 steps/day on 7 days/week. Women had a lower probability of achieving 30 min/day MVPA on 5 days/week (OR: 0.40), or a weekly total of 150 min or 500 MET.min MVPA or 75 min of vigorous activity compared to men (OR: 0.27-0.46). In addition, they were 50% less likely to meet the guidelines for weight maintenance. The odds of engaging in 150 min/week MVPA or attaining a PAL of 1.75 was lower with higher age. Educational level was positively related with accumulating 75 min/week of vigorous activity, but negatively with taking 10000 steps/day. Smokers were 60% less likely to participate weekly in 150 min of MVPA compared to non-smokers.

**Conclusions:**

The prevalence of sufficient physical activity differed greatly depending on the definition used. Women and subjects older than 35 were less likely to meet the guidelines than men and younger subjects and thus are important groups to target in future interventions.

## Background

Consistent evidence has confirmed that regular physical activity is associated with numerous health benefits and a reduced risk of several chronic diseases [1,2]. Thus, increasing activity levels has become a public health priority and has led to the publication of various physical activity guidelines over the past decades. In 1995, the Centers for Disease Control and Prevention and the American College of Sports Medicine (ACSM) recommended that “every adult should accumulate 30 min or more of moderate-intensity physical activity on most, preferably all, days of the week” [3]. These recommendations were updated by ACSM and the American Heart Association (AHA) in 2007 and stated that “all healthy adults need moderate-intensity aerobic activity for a minimum of 30 min on five days each week or vigorous-intensity aerobic activity for a minimum of 20 min on three days each week” [4]. These guidelines are largely similar to the 1995 recommendations, but they also incorporated vigorous-intensity activity and emphasized the additional benefits of physical activity beyond the minimum amount. Furthermore, they explicitly added that activity should be accumulated in bouts of at least 10 min. One year later, the US Department of Health and Human Services issued the Physical Activity Guidelines for Americans, in which it was recommended that adults should participate weekly in at least 150 min of moderate-intensity or 75 min of vigorous-intensity aerobic activity or an equivalent combination of both [5]. The shift in focus from a minimum frequency requirement to a total weekly amount provided important flexibility to accumulate activity across the week [6]. However, the report still notes that activity should be performed in bouts of at least 10 min and should preferably be spread throughout the week. At the same time, they increased the guideline for vigorous activity from 60 to 75 min/week and underlined that greater amounts of activity may confer additional health benefits. Although there are no official recommendations for the European Union [6], both the World Health Organization and the British Association of Sport and Exercise Sciences have confirmed the recent US guidelines [7,8].

It is important to note that the previous guidelines are focused towards improving overall health and reducing the risk of several chronic diseases. However, these activity levels might be insufficient to maintain a healthy body weight. Several organizations have declared that adults should attain a physical activity level (PAL) of 1.75 or more to prevent excessive weight gain and avoid the transition to overweight or obesity [9-11]. In addition, it should be noted that the recommended amounts of physical activity in the current guidelines are built upon self-reported data, which might not be directly comparable to objective measures of physical activity. One recommendation that was explicitly made for objectively measured physical activity is the guideline of 10000 steps/day, which is widely familiar to the media and general public [12,13].

Numerous studies have examined whether adults were meeting a specific guideline [14-18], but few have compared the compliance with several physical activity guidelines within the same sample [19-21]. However, since some of the guidelines are more stringent than others, compliance rates may differ according to the definition used. In addition, levels and patterns of physical activity may vary as a function of gender, age, educational level, socio-economic status, marital status, etc. [17,20,22].

Most of the previous studies have relied on self-reports and often focused on leisure time physical activity (LTPA) [14,20,23,24]. However, these measures ignore everyday activities performed for the purpose of work, transport or household chores. Recently, accelerometers have been used more often to objectively assess physical activity across the day and thus provide a more accurate picture of total physical activity. Nevertheless, most studies were limited to a minimum wear-time of 10 hours/day for 1–4 days/week [15-17,25]. However, when evaluating compliance with recommended amounts of physical activity per week, it becomes imperative to monitor activity during all waking hours for the entire seven days. Moreover, accelerometers, typically worn on the hip, are unable to detect cycling, static work, isolated arm movements, carrying loads, or locomotion on a gradient [26,27]. By combining accelerometry with physiological sensors, the SenseWear can detect small increases in energy expenditure associated with everyday activities [28,29]. In addition, the SenseWear device has the potential to measure physical activity over a full 24-hour period for seven consecutive days.

The purpose of the present study was to evaluate compliance with different physical activity guidelines in Flemish men and women, as measured with the SenseWear Armband. Furthermore, associations between meeting the guidelines and socio-demographic characteristics were examined. It was hypothesized that the prevalence of recommended amounts of physical activity would differ greatly according to the guideline used.

## Methods

### Subjects

Subjects were recruited from various companies and different work sectors (private companies, multinationals, education, research, social and welfare services, municipal services and industry) in Flanders, Belgium. Individuals volunteered to participate in the study and provided informed consent prior to participation. The study was approved by the Medical Ethics Committee of the KU Leuven. A total of 442 subjects (212 men and 230 women) between 22 and 64 years (41.4 ± 9.8 years) were enrolled. Subjects did not receive any financial reimbursement for participating in the study. However, they knew that they would be given a detailed activity and health report afterwards.

### Physical activity

Subjects were asked to wear a SenseWear Pro 3 Armband (BodyMedia, Inc., Pittsburgh, PA, USA) 24 hours a day except during water-based activities, for seven consecutive days [30]. The SenseWear is a multisensor body monitor, worn over the triceps muscle of the right arm. It enables continuous collection of various physiological and movement parameters through multiple sensors, including a two-axis accelerometer and sensors measuring heat flux, galvanic skin response, skin temperature and near body ambient temperature. Data from these sensors are combined with gender, age, body weight and height, to estimate energy expenditure, physical activity intensity and number of steps, using algorithms developed by the manufacturer (SenseWear Professional software, version 6.1). Body weight and height were measured by trained staff with subjects barefoot and in underwear. Previous studies have shown that the SenseWear tends to overestimate energy expenditure of moderate-intensity activities and underestimate that of very vigorous activities, largely due to a ceiling effect at an intensity of 10 MET [31,32].

In addition, subjects were asked to register their activities in an electronic diary, each time a new activity was started, for the entire seven-day period. The diary software program was developed at the Department of Kinesiology of the KU Leuven and stored in a Palm Z22 Personal Digital Assistant (Palm, Inc., Sunnyvale, CA, USA). The diary consisted of seven categories: sleeping/resting, personal care, eating/drinking, job, leisure time, transport and household chores. The last three categories were divided into a number of subcategories, to allow subjects to specify their activity in more detail. For this study, information from the diary was used to substitute missing SenseWear data, due to removal of the Armband. Missing values for sleep were imputed with the mean metabolic equivalent (MET) value of observed sleep during all other nights. Missing data of personal care and swimming were substituted with a constant MET-value according to the Compendium of Ainsworth (2 and 6 MET, respectively) [33]. In 50% of the days, less than 13 min were imputed based on the information from the diary.

This study included the results from participants with seven valid monitoring days. A valid day was considered a day with at least 1368 min of data, after imputation of known activities, which corresponds to 95% of a 24-hour period.

Several parameters were calculated from the SenseWear data. Time spent in moderate-to-vigorous physical activity (MVPA) and vigorous physical activity was calculated from periods of continued physical activity. Ten-min bouts were defined as 10 or more consecutive minutes with a MET-value ≥3 and ≥6, respectively [4]. Furthermore, to allow comparison with previous studies, modified 10-min bouts were defined as 10 or more consecutive minutes above the respective MET-value, with acceptance of interruptions of 1 or 2 min below the threshold [16]. Total daily time (min/day) of MVPA and vigorous physical activity, accumulated in (modified) 10-min bouts, were calculated for all seven days and summed over the entire week (min/week). In addition, active energy expenditure (MET.min/week) was calculated by summing minute-by-minute MET-values during bouts of MVPA. Physical activity level (PAL, expressed in MET) is an indicator of total energy expenditure, calculated as the average of SenseWear METs over the entire week. Finally, steps per minute were summed to obtain the total number of steps per day.

### Definition of the physical activity guidelines

The proportion of adults meeting the recommended amounts of physical activity was calculated using the following guidelines. Guidelines 1–7 were based on time spent in (modified) 10-min bouts.

1) 5*30 min/day MVPA: ≥30 min MVPA per day on ≥5 days/week

2) 3*20 min/day vigorous physical activity: ≥20 min vigorous activity per day on ≥3 days/week

3) ACSM/AHA 2007: meeting either of the two previous guidelines

4) 150 min/week MVPA: a weekly total of ≥150 min MVPA

5) 75 min/week vigorous physical activity: a weekly total of ≥75 min vigorous activity

6) US 2008 guidelines: meeting either of the two previous guidelines

7) 500 MET.min/week MVPA: ≥500 MET.min/week, accumulated during bouts of MVPA

8) PAL 1.75: a weekly average PAL of ≥1.75

9) 7*10000 steps/day: 7 days with ≥10000 steps/day

### Socio-demographic characteristics

Participants were categorized by gender, age (20–34, 35–49 and 50–65 years), educational level (high school or less versus college or university degree), smoking status (current smoker or quit less than six months ago versus not current smoker) and marital status (single, married/co-habiting/having a partner and divorced/widowed).

### Statistical analyses

Descriptive statistics (means and standard deviations) were presented for all physical activity variables. The prevalence of recommended amounts of physical activity was calculated for men and women separately. Logistic regressions examined the associations between socio-demographic characteristics and the odds of meeting the different guidelines. Multivariate models included age-group, educational level, smoking status and marital status. Odds ratios (OR) and 95% confidence intervals were calculated against the reference groups of males, subjects aged 20–34 years, those with less than a college or university degree, non-smokers and those being single (OR: 1.00). All analyses were performed using the SAS statistical program, version 9.2 (SAS Institute, Cary, NC, USA). Statistical significance was set at *P* < 0.05.

## Results

Characteristics of the study participants are presented in Table 1. Adherence to the study protocol was very high, with 80.8% of all subjects having valid SenseWear data for seven consecutive days. The final sample consisted of 172 men (mean age: 42.0 ± 8.9 years) and 185 women (mean age: 41.9 ± 10.3 years).

**Table 1:** Descriptive statistics for the male and female participants

	**Men (n = 172)**	**Women (n = 185)**
Age (n (%))		
20-34 years	34 (19.8%)	54 (29.2%)
35-49 years	99 (57.6%)	84 (45.4%)
50-65 years	39 (22.7%)	47 (25.4%)
Educational level (n (%))		
College or university	57 (33.1%)	44 (23.8%)
High school or less	115 (66.9%)	141 (76.2%)
Smoking status (n (%))		
Non-smokers	153 (88.9%)	147 (80.8%)
Smokers	19 (11.1%)	35 (19.2%)
Marital status (n (%))		
Single	16 (9.3%)	15 (8.2%)
Married/cohabiting/with partner	151 (87.8%)	147 (79.9%)
Widowed/divorced	5 (2.9%)	22 (11.9%)
Physical activity (mean ± SD)		
Time spent in MVPA (≥3 MET) (min/day)	91.3 ± 69.8	55.6 ± 50.4
Time spent in vigorous PA (≥6 MET) (min/day)	8.2 ± 13.3	4.1 ± 7.6
EE during MVPA (≥3 MET) (MET.min/day)	432.1 ± 331.4	249.3 ± 220.9
Physical activity level (MET)	1.66 ± 0.26	1.58 ± 0.23
Steps (n/day)	12327 ± 4182	12056 ± 3356

MVPA: moderate-to-vigorous physical activity accumulated in 10-min bouts, PA: physical activity accumulated in 10-min bouts, EE: energy expenditure.

Overall, the prevalence of recommended amounts of physical activity was higher among men than women (Table 2). According to the ACSM/AHA guidelines and using the strict definition of a 10-min bout (i.e. without interruptions), 57.6% of men and 37.3% of women met the guidelines for moderate activity, whereas only 12.8% and 7.0% met the guidelines for vigorous activity. When applying the modified 10-min bout definition, compliance rates increased to 73.8% and 55.7% for moderate activity and 13.4% and 7.6% for vigorous activity.

**Table 2:** Prevalence (n (%)) of sufficient physical activity according to the different guidelines

	**Men**	**Women**
**10-min bouts**		
ACSM/AHA 2007 ^a^	101 (58.7%)	70 (37.8%)
5*30 min/day MVPA	99 (57.6%)	69 (37.3%)
3*20 min/day vigorous PA	22 (12.8%)	13 (7.0%)
US 2008 guidelines ^b^	151 (87.8%)	130 (70.3%)
150 min/week MVPA	150 (87.2%)	126 (68.1%)
75 min/week vigorous PA	48 (27.9%)	29 (15.7%)
500 MET.min/week MVPA	154 (89.5%)	143 (77.3%)
**Modified 10-min bouts**		
ACSM/AHA 2007 ^a^	127 (73.8%)	103 (55.7%)
5*30 min/day MVPA	127 (73.8%)	103 (55.7%)
3*20 min/day vigorous PA	23 (13.4%)	14 (7.6%)
US 2008 guidelines ^b^	162 (94.2%)	161 (87.0%)
150 min/week MVPA	162 (94.2%)	160 (86.5%)
75 min/week vigorous PA	49 (28.5%)	33 (17.8%)
500 MET.min/week MVPA	164 (95.4%)	167 (90.3%)
PAL 1.75 ^c^	60 (34.9%)	40 (21.6%)
7*10000 steps/day ^d^	28 (16.3%)	26 (14.1%)

^a^ Meeting either ≥30 min/day of moderate-to-vigorous physical activity (MVPA) on ≥5 days/week or ≥20 min/day of vigorous physical activity (PA) on ≥3 days/week. Note that these 2 categories are not mutually exclusive.

^b^ Meeting either ≥150 min/week of moderate-to-vigorous physical activity or ≥75 min/week of vigorous physical activity. Note that these 2 categories are not mutually exclusive.

^c^ Average physical activity level of ≥1.75 MET.

^d^ Taking 10000 steps/day on 7 days/week.

Furthermore, 87.2% of men and 68.1% of women accumulated at least 150 min MVPA per week in 10-min bouts and thus met the criteria for compliance with the US 2008 guidelines. The proportion of adults meeting the minimum levels of vigorous activity was much lower, with only 27.9% of men and 15.7% of women achieving at least 75 min of vigorous physical activity per week. In addition, 89.5% of men and 77.3% of women expended at least 500 MET.min/week in physical activity of at least moderate intensity. Using the modified 10-min bout definition, 94.2% of men and 86.5% of women were classified as moderately active and 28.5% and 17.8% as vigorously active. Additionally, 95.4% of men and 90.3% of women met the energy expenditure criteria.

When considering the guidelines for weight maintenance (average PAL of 1.75) a weekly average PAL of ≥1.75, 34.9% of men and 21.6% of women were sufficiently active to avoid excessive weight gain.

Despite the high daily average, only 16.3% men and 14.1% of women took 10000 steps/day on seven consecutive days. However, when the frequency requirement was decreased to 5 days/week, 45.4% of men and 55.1% of women were compliant (results not shown).

The results of the logistic regressions for the association between socio-demographic characteristics and meeting the different guidelines are shown in Table 3. For these analyses, only the strict definition of a 10-min bout was used. However, OR for modified 10-min bouts yielded very comparable results (results not shown).

**Table 3:** **Adjusted odds ratios (95% confidence interval)**^**$**^**for meeting the different physical activity guidelines by socio-demographic characteristics**

	5*30 min/day MVPA		3*20 min/day vigorous PA		**150 min/week MVPA**		**75 min/week vigorous PA**		**500 MET.min/week MVPA**		**PAL 1.75**		7*10000 steps/day	
Gender														
Men	1.00		1.00		1.00		1.00		1.00		1.00		1.00	
Women	**0.40**	**(0.25-0.63)**	0.49	(0.23-1.06)	**0.27**	**(0.15-0.49)**	**0.46**	**(0.26-0.81)**	**0.33**	**(0.17-0.62)**	**0.50**	**(0.30-0.83)**	1.01	(0.54-1.88)
Age														
20-34 years	1.00		1.00		1.00		1.00		1.00		1.00		1.00	
35-49 years	0.59	(0.34-1.02)	1.39	(0.51-3.78)	**0.28**	**(0.13-0.62)**	1.89	(0.93-3.87)	**0.28**	**(0.12-0.68)**	**0.48**	**(0.27-0.86)**	0.66	(0.32-1.34)
50-65 years	0.76	(0.40-1.44)	1.53	(0.49-4.76)	**0.25**	**(0.11-0.60)**	1.01	(0.42-2.43)	0.39	(0.14-1.04)	**0.28**	**(0.14-0.60)**	0.51	(0.21-1.24)
Educational level														
College or university	1.00		1.00		1.00		1.00		1.00		1.00		1.00	
High school or less	0.76	(0.47-1.24)	0.37	(0.14-1.01)	0.66	(0.37-1.19)	**0.35**	**(0.17-0.70)**	0.63	(0.34-1.18)	1.64	(0.97-2.80)	**3.70**	**(1.98-6.91)**
Smoking status														
Non-smokers	1.00		1.00		1.00		1.00		1.00		1.00		1.00	
Smokers	0.85	(0.46-1.58)	0.40	(0.09-1.78)	**0.40**	**(0.20-0.80)**	0.43	(0.16-1.15)	0.62	(0.29-1.33)	0.70	(0.34-1.45)	1.01	(0.45-2.27)
Marital status														
Single	1.00		1.00		1.00		1.00		1.00		1.00		1.00	
Married/cohabiting/with partner	0.75	(0.34-1.68)	0.88	(0.24-3.29)	0.62	(0.19-2.01)	0.84	(0.32-2.20)	0.40	(0.09-1.80)	2.12	(0.80-5.61)	0.89	(0.31-2.58)
Widowed/divorced	0.99	(0.33-3.01)	1.96	(0.35-10.93)	1.51	(0.33-6.95)	1.55	(0.41-5.90)	0.79	(0.13-5.00)	2.00	(0.52-7.71)	0.57	(0.11-2.88)

^$^ Multivariate models included gender, age, education level, smoking status and marital status.

MVPA: moderate-to-vigorous physical activity accumulated in 10-min bouts, PA: physical activity accumulated in 10-min bouts.

Compared to men, women were significantly less likely to be classified as sufficiently active according to the ACSM/AHA guideline for MVPA (OR: 0.40) and the US guidelines for MVPA (OR: 0.27), vigorous physical activity (OR: 0.46) and energy expenditure (OR: 0.33). In addition, women were 50% less likely to meet the criteria for weight maintenance.

The odds of engaging in 150 min MVPA per week or achieving a PAL of 1.75 was significantly lower with higher age, with those aged 35–49 years being 72% and 52% less likely and those aged 50–65 years 75% and 72% less likely to meet the respective guideline compared to those in the youngest age-group. Additionally, 35–49 year-olds had a 3.6-fold reduced likelihood of accumulating 500 MET.min/week in MVPA than 20–34 year-olds.

Subjects with the lowest educational level had a significant lower probability of engaging in 75 min of vigorous physical activity per week (OR: 0.35), and a significantly higher probability of taking 10000 steps/day compared to subjects with a college or university degree (OR: 3.70).

Finally, smokers were 2.5 times less likely to participate weekly in 150 min of MVPA compared to non-smokers. Marital status was not related to meeting the different guidelines.

## Discussion

Several public health guidelines concerning physical activity have been published in the last decades. Although results may vary depending on the definition used, most studies have only applied one criterion to assess the proportion of adults being sufficiently active. This study is one of the first to simultaneously analyze compliance with different physical activity guidelines, using an objective measure of physical activity. In addition, we investigated the relationship between socio-demographic characteristics and the odds of meeting the different guidelines in order to identify groups that are currently inactive and would thus derive substantial benefits from increasing their physical activity.

As expected, the prevalence of sufficient activity varied according to the physical activity guideline and decreased as the recommendations became more stringent [19]. According to the ACSM/AHA guidelines, 73.8% of men and 55.7% of women were classified as moderately active and only 13.4% and 7.6% as vigorously active, when modified 10-min bouts were considered.

Previous studies have also shown that compliance rates were lower for vigorous physical activity as compared to moderate physical activity [14,18,23]. However, these studies used self-reports of physical activity and primarily focused on leisure time. Recently, accelerometers have been used more often to objectively quantify physical activity in daily life and evaluate the compliance with public health guidelines. However, most of these studies were restricted to recommendations for MVPA. The proportion of adults attaining at least 30 min MVPA per day from modified 10-min bouts varied from 1% in Swedish men and women [17], to 3.8% and 3.2% in men and women from the US [16] and 4.2-9.3% in Portuguese men and women [15].

Recent reports, such as the 2008 Physical Activity Guidelines for Americans and the Global Recommendations on Physical Activity for Health, focus on total volume of physical activity rather than a minimum number of sessions per week [5,8]. Thus, individuals can achieve recommended amounts of activity in a number of different ways. For example, weekend warriors may participate in one or two bouts of exercise to meet these goals. Nonetheless, the US guidelines add that physical activity should preferably be spread throughout the week [5].

In the current sample, 94.2% of men and 86.5% of women accumulated ≥150 min MVPA per week, but only 73.8% and 55.7% did so in five or more sessions of ≥30 min/day. Thus, removing the frequency and duration requirements resulted in a considerable increase in the prevalence of sufficient MVPA. Furthermore, despite the upward shift from 60 to 75 min/week, the proportion of adults meeting the minimum levels of vigorous activity was higher with the current than with the ACSM/AHA guidelines.

The less restrictive nature of the recent guidelines is demonstrated in several previous studies. Carlson et al. [20] examined the prevalence of LTPA and reported that 34.8% and 30.5% of US men and women were considered active according to the ACSM/AHA guidelines, compared with 47.4% and 39.9% using the US 2008 guidelines. Reasons for the higher percentages were the removal of the frequency and duration requirements but also the possibility of combining moderate and vigorous physical activity. In addition, Rafferty et al. [34] demonstrated that 40.2% of men and 36.1% of women who reported walking in their leisure time, walked for a total of 150 min/week, but only 23.5% and 20.0% walked for at least 30 min/day five or more times per week.

The prevalence of objectively-measured physical activity is much lower, but also indicates the difference between both guidelines [21]. In addition, Tucker et al. [25] noted that compliance with the US guidelines varied considerably according to the criteria used. When considering time spent in MVPA, 9.5% of men and 7.0% of women achieved the recommended minimum. In contrast, using the energy expenditure criteria (≥500 MET.min/week), 57.7% of men and 32.5% of women were classified as sufficiently active. However, energy expenditure was calculated by accumulating accelerometer MET-minutes individually, whereas only periods of 8–10 consecutive minutes were considered for time spent in MVPA.

It is important to note that most of the previous studies defined a 10-min bout as 10 or more consecutive minutes of at least moderate intensity, with allowance for 1–2 min below the threshold [15,16,21,25]. In 1995 it was indeed noted that bouts of physical activity as short as 8–10 min provided beneficial health and fitness effects [3]. However, since 2007, the reports state that activity should be accumulated from bouts lasting 10 or more minutes [4,5,8]. Therefore, it seems more appropriate to use a strict definition of a 10-min bout, i.e. without any interruptions. When we applied this criterion the prevalence of sufficient physical activity decreased, especially for moderate activity and to a smaller extent for vigorous activity. Moderate-intensity activities may represent activities of daily living, which are intermittent in nature, whereas vigorous activity is probably performed for exercise intentions and thus more continuous.

Nevertheless, even when using the strict definition of a 10-min bout, the proportion of adults meeting the different guidelines was relatively high compared to previous studies. However, making comparisons between studies is difficult and several factors might explain the observed differences. First, various instruments were used to measure physical activity. Accelerometers accurately assess ambulatory activities, but may underestimate overall physical activity, due to the inability of detecting cycling, upper body movement, carrying loads or walking on an incline [26,27]. In contrast, by combining accelerometry with physiological sensors, the SenseWear may capture the additional energy-cost of these lifestyle activities [28,29]. In addition, the use of the electronic diary allowed us to impute missing data for swimming. Secondly, methodological differences in collecting and analyzing data make results difficult to compare. For example, estimates of time spent in MVPA may differ substantially according to the cut-points used. Hagströmer et al. [17] showed that, when using cut-points derived from both ambulatory and non-ambulatory activities, 95% of the sample achieved 30 min/day of MVPA, compared to 52% when applying cut-points based on ambulatory activities only. Moreover, estimates of adherence in previous studies were frequently based on data from participants with 1–4 valid monitoring days [15-17,25]. In contrast, the current study required seven valid days to be included in analyses. In addition, physical activity was assessed over a 24-hour period, whereas the minimum wear-time in previous studies was limited to ≥10 hours/day. Thus, during some of the waking hours, activities were not registered. Another factor that limits the comparison of results is the difference in the interpretation of compliance with the physical activity guidelines. Finally, most studies used random sampling techniques, while the present study consisted of a group of healthy volunteers. Accordingly, our sample may present a more active group of adults, compared to the general population.

It should be noted that the recommended amounts of physical activity in the current guidelines are based on associations between self-reported physical activity and health outcomes [16]. Perhaps, these guidelines are not directly translatable to objective measures of physical activity. Troiano et al. [16] suggested that less than 30 min of objectively-measured physical activity may be needed to achieve substantial health benefits. However, other studies noticed that, since these self-reports primarily captured LTPA, the current recommendations should be viewed as the minimum level of physical activity over and above the routine activities of daily living [22,35]. Thus, when evaluating physical activity across the day, a higher cut-point for sufficient activity would be more suited. Several authors have proposed a cut-point for health-enhancing physical activity (HEPA) of 3000 MET.min of MVPA accumulated over 7 days or 1500 MET.min of vigorous activity accumulated over 3 days or more [22,35,36].

When we applied this cut-point, 43.0% of men and 17.8% of women were considered active (results not shown). These numbers are comparable to previous studies that used the IPAQ. Bergman et al. [22] reported that in a sample of Swedish adults, 33.5% of men and 19.1% of women reached the high physical activity category. Other studies investigated the prevalence of physical activity across countries and reported that, in Belgium, 29.6-37.2% of men and 20.5-21.9% of women met the recommended amounts of HEPA [35,36].

Another issue that needs to be addressed is whether the requirement of continuous bouts is similar for objectively measured physical activity. In a previous accelerometer study it was shown that although 52% of adults accumulated at least 30 min/day of MVPA, only 1% achieved those minutes from three or more continuous bouts of at least 10 minutes [17].

Clearly, previous recommendations should be reconsidered based on the associations between objectively measured physical activity and health outcomes.

One recommendation that was specifically created for objective measures of physical activity is the guideline of 10000 steps/day. This guideline represents 30 min of MVPA in addition to a minimum level of baseline physical activity. It has been suggested that 30 min of moderate activity translate to 3000–4000 steps, at a stepping rate of 100 steps/min [12,13,37]. Adding this amount to an estimated minimum of 6000–7000 steps, taken during the routine activities of daily living, approximates the proposed 10000 steps/day [12].

The current study showed that 16.3% men and 14.1% of women met the guidelines of ≥10000 steps/day on seven consecutive days. However, when the frequency requirement was decreased to 5 days/week, 45.4% of men and 55.1% of women were compliant. Most studies did not use the criterion of 10000 steps/day for a specific number of days, but simply looked at the average daily value, calculated from all valid days. The prevalence of adherence among adults ranged from 13.9-16% in samples from the US [38,39], to 34.5% in Canada [21] and 41.6% in Belgium [40]. In addition, Chastin et al. [19] reported that 53% of a group of UK-based postal workers achieved the recommended minimum of 10000 steps/day on at least 5 days/week.

Furthermore, the present findings show that, despite the high compliance with the ACSM/AHA and US guidelines, only 34.9% of men and 21.6% of women attained an average PAL of 1.75. To increase PAL, a high level of physical activity throughout the day would be required. This explains why individuals who accumulate ≥30 min/day of MVPA but are otherwise sedentary may meet the guidelines for cardiovascular health, without achieving the minimum levels to avoid excessive weight gain. Thus, in the light of the current obesity epidemic continued interventions to increase physical activity are needed.

This study also identified socio-demographic characteristics that are associated with meeting the guidelines and should be considered for the planning of future interventions. Similar to what is typically reported, the prevalence of sufficient physical activity was higher among men than women, irrespective of the guideline used [20,21,23-25]. Furthermore, the findings that the likelihood of meeting the physical activity recommendations was lower among women and decreased with age, are consistent with several previous studies. However, the results are not directly comparable because of discrepancies in the definition of sufficient physical activity.

Bryan et al. [23] investigated the prevalence of LTPA among Canadian adults and reported that the probability of achieving ≥30 min MVPA or ≥20 min vigorous physical activity on 4 days/week was higher for men than women in 1994–1995 and 1998–1999. But, this difference disappeared around 2001. In addition, it was shown that women were less likely to meet the HEPA-criterion of 3000 MET.min MVPA or 1500 MET.min vigorous activity per week [22,35].

Furthermore, several studies showed that the odds of engaging in sufficient physical activity decreased with increasing age [18,23,35]. Bergman et al. [22] demonstrated that those being younger than 55 were 1.5-1.7 times more likely to be classified as active according to the ACSM/AHA guidelines in crude analyses. However, after adjustment for other socio-demographic characteristics, these associations were no longer significant. Nevertheless, 18–34 year-olds had a 1.8-fold higher odds of reaching the HEPA cut-points than 55–74 year-olds, after adjustment for all socio-demographic correlates.

The present findings also demonstrated that physical activity patterns differed between educational groups. Subjects with the lowest educational level had a significantly lower probability of obtaining 75 min of vigorous activity per week compared to those with a college or university degree. This may reflect the lower probability of participating in LTPA, as indicated by several previous studies [41,42]. In contrast, lower-educated individuals may have more physically demanding jobs that require a large amount of ambulatory activity. This might explain why the lower-educated were almost 4 times as likely to take 10000 steps/day.

Our results confirm those of earlier studies. Macera et al. [18] showed that the odds of accumulating 30 min of moderate-intensity activity on 5 days or 20 min of vigorous activity on 3 days/week during non-working hours increased with educational level. Additionally, the proportion of adults meeting the ACSM/AHA or US guidelines was higher with greater educational attainment [20,24]. In contrast, Bergman et al. [22] showed that education was not related to meeting the ACSM/AHA guidelines, but that subjects with a college/university degree were less likely to meet the HEPA-criterion than those with basic education.

With regard to steps, Chastin et al. [19] demonstrated that 77% of delivery postal workers met the minimum of 10000 steps/day on 5 days/week, compared to only 28% of the office-based postal workers. Furthermore, it was shown that the number of steps/day on weekdays differed between occupational groups with professionals and managers recording the lowest (7883 steps) and blue collar workers the highest number of steps (11784 steps). The difference of almost 4000 steps/day suggest that those with the highest occupational status would have to walk for an additional 30 min during non-working hours to reach the steps of those with a physically active job [43].

In addition, it has been suggested that factors of an unhealthy lifestyle such as smoking and being physical inactive tend to cluster, which is consistent with the present observations [41,44,45]. Smokers had a 60% reduced likelihood of participating weekly in 150 min MVPA compared to non-smokers. Similarly, Bertrais et al. [14] demonstrated that current smokers were 24-27% less likely to achieve 150 min MVPA or 60 min vigorous physical activity per week.

Finally, in accordance with the present results, Bergman et al. [22] showed that after adjustment for other socio-demographic correlates, marital status was not related to meeting the ASCM/AHA or HEPA guidelines. However, in gender-specific analyses, being single was positively associated with achieving the HEPA guideline with single women being twice as likely to meet this goal compared to women who were married or co-habited.

A major strength of this study was the use of a valid activity monitor to objectively assess physical activity across the day. Furthermore, participants were asked to wear the monitor 24 hours a day and only participants with at least 22 hours and 48 min (95% of 24 hours) of data for seven consecutive days were included in the analyses. Less than seven days may be enough to assess habitual physical activity [30], but when evaluating compliance with recommended amounts of physical activity per week, it is stronger to use seven days rather than estimating the prevalence of compliance based on data from participants with one or more valid monitoring days [19]. On the other hand, it has been suggested that compliance rates could be overestimated by excluding those who did not wear the monitor for seven days, because the least active tend to be less compliant with the study protocol [46]. However, 81% of the current sample wore the monitor for seven consecutive days and physical activity levels did not differ between those with and without seven valid monitoring days.

However, some limitations should be recognized. As previously stated, participants volunteered to engage in the study. This may have led to a selection bias because subjects who agreed to participate may have been more active than the general Flemish population. Accordingly, the generalizability of these findings may be restricted. In addition, subjects knew they participated in a physical activity study and were monitored for their activity. Thus, because of a possible Hawthorne effect, participants could have performed more physical activity than usual. However, it was not our aim to describe habitual physical activity in the general population, but rather to compare different activity guidelines within the same sample. Secondly, similar to other activity monitors, the SenseWear is known to overestimate energy expenditure of moderate-intensity activities and underestimate that of very vigorous activities, mainly due to a ceiling effect at 10 MET [31,32]. However, this would not affect the estimate of time spent in vigorous activity, since the threshold was set at 6 MET. Nevertheless, Berntsen et al. [32] showed that time spent in MVPA was overestimated by both the Actigraph and SenseWear compared to indirect calorimetry (2.5% and 2.9%, respectively). In addition, Dwyer et al. [47] reported that during treadmill walking, the average SenseWear step count was 5% less than the manual count, which is similar to other motion sensors and pedometers, designed specifically to measure steps [48].

## Conclusions

The prevalence of sufficient physical activity differed greatly depending on the definition used. 87.2% of men and 68.1% of women achieved 150 min of MVPA per week, but only 57.6% and 37.3% accumulated this amount as at least 30 min/day on five or more days per week. The proportion of adults meeting the recommended amounts of vigorous physical activity was considerably lower, with 27.9% of men and 15.7% of women achieving 75 min/week and 12.8% and 7.0% achieving 20 min/day on three or more days per week. In addition, 34.9% of men and 21.6% of women were sufficiently active to avoid excessive weight gain. The lowest compliance rates were observed for the goal of 10000 steps/day. These results highlight the need to further examine the nature of the different guidelines and determine the most appropriate way to communicate physical activity requirements to the general public.

Women and subjects older than 35 were less likely to achieve the recommended amounts of physical activity and should thus be targeted in public health interventions designed to increase physical activity. The use of unobtrusive objective instruments, like the SenseWear Armband, may help people to monitor their activity levels and find ways to increase their activity to an amount that would provide substantial health benefits.

## Competing interests

The authors declare that they have no competing interests.

## Authors’ contributions

TS contributed to the design of the study, collected, analyzed and interpreted the data and drafted the manuscript; RP participated in the coordination of the study and revised the manuscript critically for intellectual content. JL conceived the study, helped with statistical analyses and interpretation of the data, revised the manuscript and had general supervision of the study. All authors critically read and approved the final manuscript.

## Pre-publication history

The pre-publication history for this paper can be accessed here:

http://www.biomedcentral.com/1471-2458/13/136/prepub
